# The Fourth Industrial Revolution – Smart Technology, Artificial Intelligence, Robotics and Algorithms: Industrial Psychologists in Future Workplaces

**DOI:** 10.3389/frai.2022.913168

**Published:** 2022-07-06

**Authors:** Rudolf M. Oosthuizen

**Affiliations:** Department of Industrial and Organisational Psychology, University of South Africa, Pretoria, South Africa

**Keywords:** fourth industrial revolution, industrial psychologist, career, change, disruptive technology, competence, STARA, strategic intelligence

## Abstract

In the Fourth Industrial Revolution (4IR), STARA (smart technology, artificial intelligence, robotics, and algorithms) is predicted to replace a third of the jobs that exist today. Almost twice as many current work tasks will be handled by robots. It is forecast that by 2025, 85 million jobs may be displaced by a shift in the division of labor between humans and machines, while 97 million new roles may emerge that are more adapted to the new division of labor between humans, machines and algorithms. Industrial psychologists are playing an increasingly important role in the workplace due to these trends from a strategic intelligence perspective. The objective of this article is to present a critical review of industrial psychologists in future workplaces in the context of the 4IR - STARA. A competence model is posed for industrial psychologists to perform a strategic intelligence role in organizations in the 4IR.

## Introduction

Industrial psychologists have a key role to play in strategic and operational human resources (personnel) practice and people (individual, group, and organizations) behavior dynamics, as well as assessment and intervention design. The industrial psychologist's strategic intelligence role is indispensible in realizing an organization's success. The value of strategic intelligence can be enhanced by improving the skills of managers and employees, which in turn improves their ability to learn about the potential 4IR changes in their organization, by allowing them to communicate freely to share their perceptions, new information, and insights whenever and wherever the organization requires such information, the “intelligence quotient” of all organizational managers and employees will be increased (Ogbeibu et al., 2021c).

Accordingly, industrial psychologists apply psychology in the workplace, provide interventions to modify poor performance and implement programmes for industrial psychology intervention (Graupner, 2021). In the human workforce, there is a mandate for advancement (Jackson, 2014). Advances in technology, and the associated benefits and drawbacks, are critical issues regarding the workforce that merit in-depth discussions. Although the workforce may undergo a transformation over a century, Elliott (2014) suggests that organizations need to understand the capabilities of technology and how it will affect employee behavior in the next 10 years or so (Chuang and Graham, 2018). The growth of smart technology, artificial intelligence (AI), robotics, and algorithms, or STARA (Bort, 2014; Lynch, 2015), has led Stephen Hawking and Bill Gates to warn that mass unemployment will result (Bort, 2014; Lynch, 2015).

STARA is estimated to eliminate 33% of occupations by 2025 (Frey and Osborne, 2013; Thibodeau, 2014) due to improvements in robotic dexterity and intelligence, combined with the development of low-cost autonomous units that can potentially outperform people in a wide variety of work settings and dynamic activities (World Economic Forum, 2020). The web of things, self-checkout systems in retail, cellphone applications, bookkeeping robotisation, and driverless vehicles are examples of these types of innovations. As a result of these types of innovation, it is impossible to imagine prolonging workers in certain positions due to cost advantages. In addition, recent debates emphasize the importance of teams to collaborate digitally and interdependently on set tasks, and for industrial psychologists to develop competencies fundamental to the STARA model, to catalyze innovation initiatives and reduce turnover expectations (Brougham and Haar, 2018; Ding, 2021; Ogbeibu et al., 2021a).

STARA is not simply being tapped into low-paying, low-talented jobs. There is greater use of high-tech algorithms in research, and information-writing algorithms are becoming more advanced in organizations and communications in general. Robots with high-precision finesse are being used increasingly as well. In an investigation of 702 professions, the likelihood of STARA claiming employment was identified. Accounting, commercial pilots, client management, sales, and office workers are among the occupations at risk (Frey and Osborne, 2013; Bhargava et al., 2021). In addition, STARA could have a substantial impact on healthcare (Bloss, 2011; Lorentziadis, 2014), education (for example, through web-based learning), transportation, and farming. In general, STARA threatens to eliminate 47% of occupations (Brougham and Haar, 2018).

South Africa's President Cyril Ramaphosa has incorporated the Fourth Industrial Revolution (4IR) into his economic strategy, provoking criticism for its neoliberal rhetoric echoing the World Economic Forum (WEF) and concern that it will not lead to job creation. Corporations must rethink their strategies and auto-cannibalize their business models. Historically, it has been considered a way for policymakers in manufacturing nations to increase national competitiveness and bring manufacturing in-house. However, it may prevent developing countries from attracting labor-intensive manufacturing that would create jobs (Hu, 2021). This could reduce the demand for low-skilled workers and increase inequality by reducing the demand for work. Due to deficiencies in the education system, South Africa has a significant skills shortage, limiting the supply of managers, researchers, and workers for 4IR (Venturini, 2022). The poor quality of infrastructure reflects poor governance and state capture. In the cybersecurity and data protection arenas, it has a poor track record in policy formulation and implementation. Despite aspirations, the domestic market is small, and access to the rest of Africa is difficult due to its limited purchasing power and poor distribution system. Moreover, South African firms must compete with strong Chinese firms (Sutherland, 2020).

### Objective of This Article

The objective of this article is to present a critical review of industrial psychologists in future workplaces in the context of the 4IR - STARA. A competence model is posed for industrial psychologists to perform a strategic intelligence role in organizations in the 4IR.

The subsequent section, the literature review focus on the emergence of digital workspaces in the fourth industrial revolution, and conceptualization of smart technology, artificial intelligence, robotics and algorithms (STARA).

## Literature Review

### The Emergence of Digital Workspaces in the Fourth Industrial Revolution

In 4IR, people, objects and systems are interconnected through the real-time exchange of data throughout the entire value chain, resulting in an increasingly digitized world. A significant characteristic of this society is the proliferation of increasingly complex technologies, which integrate the physical, digital, and biological worlds (Spath et al., 2013; Dorst et al., 2015; Rotatori et al., 2021). This interconnection leads to the advent of products, machines, and processes equipped with artificial intelligence and capable of adapting to spontaneous changes in their environment. In addition, smart technology becomes integrated into broader systems, enhancing the ability to create flexible, self-operating production systems. Smart technology and systems can be applied to a broad range of fields (Huber and Kaiser, 2015; Porter and Heppelmann, 2015; Hecklau et al., 2016).

The 4IR's core component involves autonomous production methods powered by robots, which carry out tasks intelligently while focusing on safety, flexibility, versatility, and coordination. Automation will result in job losses, which means industrial psychologists will have to assist people with coping with the loss of their jobs. The 4IR may indeed enhance access to mental-health services, even if automation can adversely affect some aspects of people's wellbeing. Two examples of the use of AI to provide mental health services are the use of Woebot, the world's first mental-health chatbot, and Tess, built by psychologists to coach people to build resilience by having text message conversations—similar to talking to a friend or coach (Gower, 2018). Integrating robots into human workspaces, however, helps it to become more economic and productive, and opens many possibilities in industries (Cheng et al., 2021). With the latest technological innovation, industrial robots are evolving to facilitate the 4IR (Roland Berger Strategy Consultants, 2014). As they work together in the 4IR, humans and robots will interlink tasks and use smart sensors to create human-machine interfaces. Various functions can now be performed by robots, including production, logistics, and office management (to distribute documents), and they can be controlled remotely.

This implies that the 4IR is based on cyber-physical systems, the Internet of Things, and the Internet of Services. There is a growing number of companies joining the movement and using various approaches to enhance competitiveness and gain productivity and economic benefits from it (Trauth-Goik, 2021). Although the 4IR covers a wide array of manufacturing applications, the trend is quickly taking shape through the emergence of robotics and automation product innovations that are specifically designed for the industrial revolution. According to Hecklau et al. (2016), the 4IR presents many opportunities for companies; however, there are also many challenges as a result of ongoing digitisation and automation.

#### Financial Challenges

As globalization continues, organizations must deal with shorter product life cycles, the need to remain competitive, and reduced time to market (Helmrich, 2015). To achieve an advanced level of service orientation, organizations must rationalize their innovation processes (Shahd and Hampe, 2015; Hecklau et al., 2016; Umar et al., 2021). Finance and process-driven roles are on the decline while roles that require thinking outside the box are gaining traction and will be in demand. However, customers must still be persuaded that technology will improve their lives. The cloud and big data are particular developments that are affecting all businesses. Customers are becoming more empowered through technology and data. The power of technology cannot be underestimated. As a result, the future pricing models and unit costs of finance will change. Cloud technology is also safe and allows updates to be completed seamlessly. Data can be accessed anywhere and anytime, which has fundamentally changed how businesses operate. Real-time reporting has also fundamentally changed how businesses operate. Business leaders can receive more information directly in the format they prefer (Nyambo, 2020).

#### Societal Challenges

Young employees need to be attracted while older employees need to be retained for their expertise. Social values among younger generations are work-life balance and balancing work with family (Stock-Homburg, 2013). A growing number of virtual jobs and flexible work topics also call for new forms of lifelong learning (Brühl, 2015). The multifaceted nature of processes is creating jobs requiring more qualifications. To qualify employees for more strategic, coordinating, and creative responsibilities, organizations must provide them with more training in these areas (Hecklau et al., 2016; Rotatori et al., 2021).

#### Technical Challenges

Big data present many challenges to companies (Huber and Kaiser, 2015). Communication networks and internet protocols are among the many information technology infrastructures that need to be built and implemented (Brühl, 2015). To facilitate cooperative work on different platforms, standardized interfaces and open architectures should be created (Shahd and Hampe, 2015). Keeping large amounts of data on external servers raises the issue of cybersecurity since unauthorized access to the data must be prevented. Further training is needed for employees to adapt to the increased use of virtual work (Hecklau et al., 2016; Ross and Maynard, 2021).

#### Ecological Challenges

Climate change is one of the most significant challenges facing the environment (Elheddad et al., 2021). All living creatures within the biosphere are affected by the continuous changes occurring within the environment. It is also increasingly important to use ecological resources efficiently since many of them are scarce. Consequently, organizations are increasingly recognizing their role in implementing sustainable solutions (Spath et al., 2013; Hecklau et al., 2016).

#### Political and Legal Challenges

Governments must support organizations in developing new technologies, as well as incorporating those technologies into the current environment. Governments should also establish legal parameters for the use of big data. While interacting with smart objects, data will be collected on each system as a result of the interaction (Brühl, 2015). Considering increasing work flexibility, policies and procedures regarding work times and safety matters must be established to protect employees (Hecklau et al., 2016). Governments could find themselves increasingly powerless against megacorporations, the Exponential Organizations. Regulating the activities of these global behemoths (and raising taxes from them) may be beyond the grasp of governments. If government agencies are too slow to adopt new technologies, they will both fail to generate the efficiency gains needed to keep public services going, and damage the reputation of government (Lye, 2017).

### Smart Technology, Artificial Intelligence, Robotics and Algorithms

Industrial psychologists are faced with one of the biggest issues of their times: What impact will the march of STARA have on either where people work or how people work in the future? Do we need to work in the future? Where do they fit in a world of automation? Automation is predicted to impact careers and the workplace in many ways, many analysts focus on smart technology. Luz Tortorella et al. (2021) point out that the real story has less to do with technology and more to do with how humans choose to use it. A complex, changing and competitive set of forces will determine the shape that the workforce of the future takes. Although some of these forces are evident, we cannot predict the pace at which they will manifest. As the transition to an automated workplace progresses, policies and laws, governments that enforce them, and consumer, employee, and citizen sentiments will all influence its fate. Careers in 2030 will be shaped by how this battle plays out (Kojm, 2012). It is impossible to predict linearly what will happen when so many factors are at play. Organizations, governments, industrial psychologists, and individuals must be prepared for a wide range of outcomes, even those that may appear unlikely (Stubbings, 2018).

#### Smart Technology

During the last few decades, wireless communication and sensing smart technologies have made it possible for smart learning environments to detect the context of the environment and quantify the attention available to an employee. Durães et al. (2018) point out that the development of smart learning environments is based on the rapid progress of wireless communication and sensing smart technologies. Computer scientists refer to a smart environment as a digitally augmented physical environment where sensor-enabled and networked devices work continuously and collaboratively to improve the standard of living for citizens (Chang and Chen, 2021). Today, smart environments are becoming a reality thanks to developments in technology such as mobile communications, wireless sensors, pervasive computing, machine learning, robotics, middleware and agent technology, and human-computer interfaces. As defined by Cook and Das (2005), the concept “smart” refers to the ability to autonomously acquire and apply knowledge, while the concept “environment” refers to an employee's surroundings.

In conjunction with this technological advancement, job opportunities have evolved, bringing about numerous and wide-ranging changes. There is a growing concern about indicators that are tarnished by changes, such as the need to react quickly to changes, which, in severe cases, can compromise the life and wellbeing of employees. When moderated, it impedes general cognitive abilities, concentration and productivity. Many of these careers are so-called desk jobs, in which people often work more than 8 h every day (Liao and Drury, 2000; Durães et al., 2018).

#### Artificial Intelligence

Digital platforms and artificial intelligence can shape and underpin the world of work in an unlimited way (Haefner et al., 2021). In this platform stratum, the value chain is digitalized, and the back office is commoditised automated. This also comes with warnings. As a trading platform can flourish, it can also take over the entire financial system, putting it at risk to cyberattacks and manipulation on a broad scale (United Nations Department of Economic Social Affairs, 2010). Digital platforms are closely linked to data. Every world—even the most human-centric—is shaped by how governments, organizations and individuals share and use data. Artificial intelligence in the form of digital assistants and machine learning (ML), a branch of AI that mimics the way humans learn, is increasing in accuracy as they use data and algorithms to imitate this process. The system could understand, learn, and act based on the information it gathered.

There are three levels of artificial intelligence. With assisted intelligence, people and organizations can enhance what they are already doing. GPS navigation software, for example, offers drivers directions and adjusts to road conditions. In the era of augmented intelligence, individuals and organizations can do things that would otherwise be impossible (Haefner et al., 2021). Shuttle services, for instance, would not exist without a combination of programmes that manage them. Intelligent machines that act independently will be developed in the future through autonomous intelligence. When they become more widely used, self-driving vehicles could be an example. Using AI to help humanity process, analyze and evaluate the massive amounts of data that create today's world, could allow mankind to spend more time engaged in creative thinking, decision-making and problem-solving (Stubbings, 2017, 2018).

The advancement of big data and technology is heavily reliant on artificial intelligence and machine learning. It is inherently multidisciplinary, which makes it difficult to understand, evaluate and exploit these technologies. It is true that most companies developing AI or machine learning with a nexus to human resources will have teams composed of engineers, computer scientists, developers, data scientists and other math- and tech-savvy people (Raisch and Krakowski, 2021). In the field of industrial psychology, AI/ML applications are on the rise. Thus, technology remains the focus, a trend likely to continue for some time. A field such as industrial psychology is more susceptible to getting lost in the shuffle during a technology-dominated environment and losing sight of the critical role that industrial psychologists can play (Putka and Dorsey, 2018).

#### Robotics

Until recently, most robots were slaves to their human operators; now they are becoming increasingly autonomous and powerful. As robots are increasingly used, the question arises of how robots can be successfully integrated into human-robot teams. According to Richards (2017a), humans and machines, or “agent” members, can share goals through delegation. The increased power and capacity of robots have caused a great deal of paranoia (Righetti and Smart, 2021). Reports in the media suggest that robots may soon usurp large segments of today's workforce, particularly in industries that already use advanced automation. This is probably a reasonable concern. The number of robots sold worldwide in 2014 increased by 29% to 229,261 units. Robots give humans the ability to withdraw from monotonous, risky, or challenging tasks (Richards, 2017a).

The advancement of robotics will pose more questions about robot-human integration as advanced robotics takes expansion to an entirely new level. Modern robot designs can become agent-based models (ABMs) that can be connected to other robots as well as to a wider network made up of humans and machines. This trend is already gaining momentum. Robots and humans work together daily in advanced space systems. Museum visitors may be accompanied by robot tour guides, and some hospitals have already used robot assistants. People, especially those who are frail or aged, will be able to receive help through ABMs soon. In advanced industrial plants, robots will increasingly work as part of a human-agent team (Tresa et al., 2021).

For a group to function well, trust must be of a specific nature. Up until now, most robots have been working as slaves under human supervision. Sources of information provided by them have been unsurprising, making it easy to understand their intentions. Those robots can easily be integrated. Yet, as the operators gain more autonomy, a human-robot relationship will need an increased level of adaptability when considering the assignment of power. People and robots could communicate more effectively if there were a formal system of control in place (Bhargava et al., 2021). Two distinct ways exist for human operators to see the robot components. As an alternative, a bottom-up methodology would mean that ABMs would continue to serve as simple machines that satisfy human objectives. Alternatively, the ABMs could be considered equivalent to individuals within the group using a top-down methodology. According to Richards (2017b), a top-down methodology would allow the elements to shape similarly to customary human groups, with characterized jobs and norms of conduct.

Eventually, as robots become more autonomous, monitoring may become necessary. Monitoring could be the responsibility of human supervisors. Although security systems may be automated to perform repetitive tasks, a human is still required to monitor their performance to ensure quality. However, a human must authorize an ABM to perform the last activity. The robot could be allowed to perform more important tasks if security were not a concern. Richards (2017b) further indicates that the individual becomes a manager of a human group during the developed phase of ABM self-rule. The implications of this are numerous. A human manager may, in general, prove to be more appealing to many workers. Additionally, Richards (2017b) states that people would usually scrutinize a manager's idea of what tasks to perform if a robot became one. It is just that other robots within the group would not suggest such discussions without specifically intending to do so (Alcover et al., 2021).

For industrial psychologists to analyze robot-human groups only from a quantitative perspective is insufficient. Human relationships will be affected by robots in groups over a prolonged period if robots are present in a group. How will this affect trust within the organization? It may be that profitability increases initially but relationships within the group change as errors are perceived as becoming routine. The findings of investigations that emerge through human-robot collaboration may eventually become less fundamental. The rise of AI could lead to robots being viewed as social specialists. There is a risk that a group can become self-contained with “limited wisdom” (Richards, 2017b).

#### Algorithms

Economic and policy makers hoped that the rise of the internet would lower labor market search costs and improve market outcomes. A design platform provides information on products and trades but in many cases, it also generates recommendations about whom to trade with or what to buy (Resnick and Varian, 1997; Adomavicius and Tuzhilin, 2005; Varian, 2010; Horton, 2017). In an algorithmic system, preferences can be inferred, the possible choices identified and then forced optimisation problems solved for the would-be buyer. Algorithmic systems can incorporate information that no single party is aware of. Furthermore, the quality of these recommendations increases with scale, and they have zero marginal cost.

At the moment, algorithmic recommendations are rare in the labor market; however, as more labor market aspects become computer-mediated, recommendations will become more valid. Nevertheless, labor market recommendations do not seem to be able to significantly improve what employers themselves can accomplish. By assessing qualities that are difficult to capture in a statistical model, industrial psychologists assist in choosing the right candidate for a particular job opening. Employers might not find it that expensive to assemble a pool of reasonable applicants. An issue with recommendations is that, from the employer's perspective, they encourage employers to give preference to some employees and ignore others. In conventional labor markets, some job search assistance programmes have shown strong crowd-out effects (Crépon et al., 2013). From a social welfare perspective, recommendation interventions are less attractive (Moser et al., 2021).

It was concluded by Horton (2017) that algorithmic recommendations can both be acted upon by employers and be effective at increasing the hiring of high-quality candidates, at least for certain kinds of job openings. Even though the algorithm functions as a “black box,” it produces recommendations strikingly similar to those that employers recruit if they do not receive these recommendations, at least within the limits of available measurements and statistical power. As such, algorithmic recommendations are a useful substitute for costly employer efforts. The relationship between job openings and workers seems superficially symmetric; job openings can easily be created and destroyed by employers at will, and workers can enter and leave the labor market, but it seems more likely that employers' decisions to create and fill a job opening are elastomeric in terms of assistance than an individual worker's participation in the labor force. Comparing the conventional market analogy with the alternative, for-profit recruiting firms offer their services mostly to businesses rather than individuals. A platform-based intervention becomes more powerful and possible as more of the labor market is mediated by computers. Platforms collect a great deal of data on market behavior and outcomes, and they have virtually complete control over the details that market participants can see and when. Those changes would have profound consequences for labor markets in terms of equity and efficiency (Tsamados et al., 2021).

### The Role of Industrial Psychologists From a Strategic Intelligence Perspective

Waghmare (2019) asserts that strategic intelligence is a highly effective source of competitive advantage since it can enhance decision making because it is based on information. It is important for industrial psychologists to focus on both people and technology in order to make the strategic intelligence process successful. Strategic intelligence is reflected in the industrial psychologist's ability to maintain reputation even when facing challenges that require critical decisions. Esmaeili (2014) proposes that strategic intelligence positively and meaningfully influences strategic decision-making and strategic planning in organizations that use intelligent systems. Additionally, the most effective factors for strategic intelligence include human resource intelligence, organizational process, technology (STARA), informational resources, financial resources, competitor intelligence, and customer intelligence (Ogbeibu et al., 2021b). Abdullah (2012) suggests that industrial psychologists need to focus on strategic leadership as a means of developing strategic intelligence. Industrial psychologists use strategic leadership to influence favorable prospects for success; but it also impacts organizational culture, resources allocation, political guidance, and consensus in the uncertain and complex global 4IR environment. Acros (2015) notes that strategic intelligence is crucial to deal with the rapid changes created by the 4IR environment, as well as adapting plans to a dynamic and changing environment.

In almost every application of intelligence, strategic intelligence serves two distinct purposes: one is for management, and the other is focused on operational and functional aspects. In strategic intelligence, questions pertaining to mission, goals, objectives, programs, and resource planning are dealt with as they pertain to management and executive functions (Waghmare, 2019). By contrast, operational intelligence is intelligence that services the needs of supervisors and line managers and focuses on the immediate, routine, and on-going activities of the frontline functions of an organization. An operation intelligence action involves identifying, targeting, detecting, and intervening (or prohibiting) illegal operations in any form. Organizational cultures need to embrace strategic intelligence.

For industrial psychologists, strategic intelligence is about having the right information at the right time to make the right decisions for the future success of their organization. The value of strategic intelligence can be seen in industrial psychologists' ability to maintain reputation even when faced with challenges that require critical decisions. Industrial psychologists are capable of identifying potential threats and changes that have taken place, and with the assistance of intelligence information at hand, can make suggestions to solve the mystery (Tham and Kim, 2002). In strategic intelligence, past and present issues are less important than the future as it looks to predict and anticipate the 4IR future and model it in a way that aligns with operations of the organization. In order for an organization to survive in a competitive market, it needs to understand its key points of improvement and the opportunities available to do so (Waghmare, 2019).

To develop insights and intelligence about future trends in 4IR, industrial psychologists can choose between functional and process approaches to strategic intelligence. An organization's strategic intelligence is often limited to isolated data sets created by individual departments, which apply their knowledge of the company's direction and strategies for success. As a result, information is rarely shared with other levels of managers within an organization, resulting in inferior decisions being made (Waghmare, 2019). Industrial psychologists can assist functionally oriented organizations to overcome barriers to sharing and utilizing strategic intelligence to shape a 4IR future. Strategic intelligence is best organized using a process-based approach. However, in some cases, such as mergers, the industrial psychologist might be required to keep the information confidential and only share it with a few executives.

An organization that develops processes to allow information sharing across business units and geographies will generally benefit from a more disseminated approach. In both approaches there are risks, but the benefits gained by the process approach are significantly greater than those gained by the functional approach. It is not an easy task to develop mature information capabilities to build a strong process approach (Waghmare, 2019). During a process approach, industrial psychologists must remain determined and focused on improving information capabilities. Due to rapid technological, structural, and disruptive changes, the strategic intelligence role of Industrial Psychologists in South Africa is of critical importance for organizations to guide them in the 4IR process. Also, Industrial Psychologists plays a pivotal role toward the understanding of the subjective “lived-through” feelings and experiences of employees and *in situ* responses to 4IR events.

The methodology applied in this study is depicted in the succeeding section in terms of the study design, eligibility criteria, data analysis, and the strategies used to ensure data quality.

## Methods

### Study Design

The critical review of the research literature entailed a broad systematic review of contemporary research on the themes of the 4IR-STARA. This approach allowed the author to evaluate documented research on the strategic intelligence role of industrial psychologists in future workplaces.

### Study Eligibility Criteria

The systematic review was limited to research published between 2015 and 2022 on documented contemporary topics in industrial psychology. EBSCOhost/Academic Search Premier and Google Scholar, an online information technology service, were used to conduct the search. The search terms used were 4IR Smart technology, Artificial intelligence, Robotics, Algorithms (STARA), strategic intelligence and industrial psychology. To identify which articles should be included or excluded from the systematic review, the full texts of publications were downloaded from the databases. Studies exploring industrial psychologists in future workplaces met the inclusion criteria for this article. The research articles were used as data sources.

### Data Analysis

A qualitative exploratory approach was used to explore the 4IR-STARA, and the strategic intelligence role of industrial psychologists in the future workplace (Cresswell, 2014). First, the author carefully read the studies to gain a better understanding of the phenomenon under investigation, 4IR-STARA and strategic intelligence role of industrial psychologists. In the second stage, the author synthesized a portrait of 4IR-STARA and the strategic intelligence role of industrial psychologists that considered its relations and connections within its aspects. The third stage consisted of theorizing about how and why these 4IR-STARA and the strategic intelligence role of industrial psychologists relationships exist as they do, and the fourth stage consisted of re-contextualizing the new knowledge about 4IR-STARA and the strategic intelligence role of industrial psychologists phenomena and relationships back into the context of how other authors have articulated the evolving knowledge. EBSCOhost/Academic Search Premier and Google Scholar academic databases were searched for relevant research published between January 2015 and January 2022 to locate 48 studies. Based on a quality assessment of publications, eight studies were identified as the primary sources of information.

### Strategies Used to Ensure Data Quality

Analytical processes that are systematic, rigorous, and auditable are among the most significant factors separating high-quality research from poor quality. As a result, the researcher articulated the findings in a way that the outcomes developed by the researcher are accessible to a critical reader, the association between actual data and the conclusions about the actual data is made explicit, and the claims related to the data set are rendered credible. In addition to the potential publication bias, consideration was also given to trustworthiness or credibility, true value and quality, appropriateness, and reflection on the research endeavor, as well as sound practice. By reviewing each article for scientific and methodological rigor and comparing them to the 4IR-STARA, and in terms of the strategic intelligence role of industrial psychologists in the future workplace, the articles' value and quality were assured. All data was retained for future review.

The findings of the study are presented in the following section in terms of Industrial psychology and the maturation of artificial intelligence and machine learning technology. A STARA competence model for industrial psychologists in the 4IR is proposed.

## Findings

### Industrial Psychology and the Maturation of Artificial Intelligence and Machine Learning Technology

Organizations manage an increasing amount of information and technology related to human resources. Increasing amounts of information are accumulating, they are becoming more sophisticated, and they are coming in a variety of forms (for example, big data). Although technological advances are emerging that can assimilate such information, they sprout faster than institutions can absorb, and faster than science can systematically assess. Organizational leaders have been racing to determine how to harness this brand-new wealth of information and technology, but in a rapidly advancing environment, it is easy to feel overwhelmed. Managers must consider difficult downstream questions when considering the value of industrial psychology beyond the publicity surrounding AI/ML human resource technology. When it comes to assessing AI/ML technology for human resource management, industrial psychology benefits leaders not only in sifting through the wheat from the chaff but also in designing a robust AI/ML human resource management system for their organization in the first place. This article poses and answers five questions about the role of industrial psychology in AI/ML assessment and creation (Putka and Dorsey, 2018).

When AI/ML technology is used to make predictions or forecasts, how does it ensure data integrity? To ensure data integrity, a person or a team must be responsible, not a machine. With regard to objectively assessing the value of 'people data' and using that data to make extrapolations, industrial psychology offers a depth and experience that exceeds many other fields. How can AI/ML technology developers verify the effectiveness of what it produces? In the context of decades-old professional principles and standards, “evidence” needs to stand up to judgement. Research and practice in the field of industrial psychology and related scientific fields provide insights into how people's psychological characteristics, behavior, and emotions can be assessed, predicted, and explained.

A developer of an application could verifyn that it will be demonstrably beneficial to an organization. Through the implementation of this technology, organizations will save 20 per cent on turnover among new employees. There is a wide variance in the quality of the proof used to support various assertions about what AI/ML can accomplish. An industrial psychologist is qualified to assess the quality of the findings and data gathered to evaluate AI/ML-related human resource technologies. What are the chances that the technology application will have unfavorable effects? It may be that an AI/ML human-resource application is worth its price if it delivers on its promises (for instance, if it reduces turnover, increases hiring speed, and increases employee engagement or competence). Organizations are reluctant to acknowledge problems, such as a reduction in employee diversity, a violation of employment laws, or a breach of employee confidentiality. An industrial psychologist has extensive experience with the trade-offs and results that result from various assessment and decision-making approaches in the employment sphere. Without understanding why the technology “works” and having subject knowledge of the content involved, it can be very hard to predict as many of these unplanned consequences.

What makes the technology work? Employment decisions do not happen in a vacuum. A multitude of regulatory environments becomes even more complex when working across jurisdictions (for example, employment and data privacy laws). Since legal requirements have generated workforce decisions, the discipline of industrial psychology has been incorporated into these matters. Industrial psychology has a fundamental role to play here. To assess its defensibility from a governing perspective, it is important to understand why the technology produces the results it does. The use of AI/ML technology has more implications than just legal implications for organizations. In technology implementation, intrinsic trust is often overlooked as a key factor. Alternatively, consider a manager in charge of promoting employees who receive assistance from a machine regarding guidance or career opportunities. The “why” behind recommendations made to managers and employees must be communicated. Research in the area of “explainable AI” is quite active but such studies can only gain from the knowledge of the theory and usage of subject-matter knowledge. Industrial psychologists are well-prepared to assist in explaining what is transpiring “below the surface” of a situation because they are educated in assessment and original theories.

Does industrial psychology lead the way in AI/ML technology change, or is it simply standing on the outside looking in, hoping to change the discussion at a later date? Putka and Dorsey (2018) note that industrial psychologists play a more prominent role when conducting AI/ML-based research and constructing sophisticated applications of the technology. Industrial psychology plays an essential role in shaping the great guarantee of AI/ML technology implementation, as well as contributing to the greater mission of the field, which is to nurture and enhance human flourishing as well as long-term business performance and sustainability.

### STARA Competence Model for Industrial Psychologists in the Fourth Industrial Revolution

STARA creates many new opportunities for organizations but at the same time, several challenges are arising from the ongoing automation and digitisation. A STARA competence model for industrial psychologists in the 4IR is proposed. The competencies are clustered into four main categories of competencies.

#### Specialized Competencies

- *STARA knowledge*: Considering industrial psychologists' cumulative task accountability, STARA knowledge is gaining importance.
- *Strategic business*: Changing from operational to more strategic functions require specialized all-encompassing competencies. The role of industrial psychologists is to increase the effectiveness of the business. Industrial psychologists must be able to add value through competitive market insights, personal capital, business influence, having the skills to get the job done, and promote agility across the entire organization (Ulrich, 2021).
- *Advances human capability*: Industrial psychologists must advance human capacity in an organization. In this role, line managers are responsible for elevating and developing talent, and for implementing human resources solutions that enhance both individual talent (human) and organizational capability (capability). Moreover, it provides a specific focus on promoting diversity, equity, and inclusion in the workplace to improve overall organizational performance (Ulrich, 2021).
- *Process comprehension*: Industrial psychologists need to have a deeper and broader understanding of process intricacy because of advanced process complexity.
- *Media abilities*: Technological and media skills are required for accumulative virtual work by industrial psychologists.
- *Programming abilities*: Digitalization and algorithms have led to a high demand for industrial psychologists who have programming skills.
- *Understanding information technology security*: Industrial psychologists need to understand cybersecurity because of virtual functions on servers and platforms (Hecklau et al., 2016).

#### Methodological Competencies

- *Simplifies complexity*: The 4IR presents many challenges for industrial psychologists, and they need to be objective when considering them. During times of uncertainty or crisis, it demonstrates an ability to distinguish signals from noise, think independently and see opportunities (Ulrich, 2021).
- *Mobilizes information*: By using 4IR technology, industrial psychologists can access, analyze, and act on data to solve problems and make informed decisions. It reflects comfort with data-driven decision-making, a keen interest in technological advancements, and a knowledgeable understanding of social issues that will impact the organization. The industrial psychologist must be able to constantly learn and adapt to the fluctuating artificial intelligence environments (Ulrich, 2021).
- *Creativity*: The need for more smart technology and innovative products, as well as for internal enhancements, calls for the creativity of industrial psychologists.
- *Innovative thinking*: Industrial psychologists could play a more active role in strategic functions, thus being innovators.
- *Problem solving*: Industrial psychologists should improve processes and procedures.
- *Conflict solving*: An advanced service emphasis increases customer associations; thus, industrial psychologists need to resolve conflicts.
- *Decision-making*: Industrial psychologists will be required to make their own decisions since they will be held more accountable for the process.
- *Diagnostic abilities*: Industrial psychologists must construct and analyze large amounts of information and multifaceted processes.
- *Proficiency assimilation*: Industrial psychologists must provide more comprehensive explanations of multiple dilemmas, such as examining increasing amounts of algorithmic data (Hecklau et al., 2016).

#### Societal Competencies

- *Intercultural abilities*: Industrial psychologists' ability to understand different cultures, especially when working internationally and nationally (Ulrich, 2021) is also important.
- *Fosters cooperation*: Industrial psychologists have demonstrated the ability to successfully facilitate teamwork and cooperation in the workplace. In addition, they are analyzed for their openness and self-awareness, as well as their ability to inspire trust and respect, build relationships and bring people together (Ulrich, 2021).
- *Language abilities*: Industrial psychologists are expected to converse and understand with international colleagues and customers.
- *Communication abilities*: As virtual work increases, industrial psychologists are required to have adequate virtual communication skills, as well as great listening and presentation abilities.
- *Networking abilities*: As a result of a highly globalized and interconnected value chain, industrial psychologists are required to take part in knowledge networks.
- *Teamwork abilities*: Industrial psychologists are called upon to respect team rules as teamwork and collective work on platforms increases.
- *Compromising and cooperative abilities*: Creating win-win scenarios in organizations with increasing project work is necessary for industrial psychologists who work alongside value chains as equal partners.
- *Knowledge transfer abilities*: Industrial psychologists could assist organizations in retaining knowledge. With the current demographic transformation, explicit knowledge and tacit knowledge must be exchanged.
- *Leadership abilities*: As responsibility increases and hierarchies flatten, every industrial psychologist becomes a leader (Hecklau et al., 2016).

#### Personal Competencies

- *Flexibility*: Industrial psychologists are more independent as virtual work expands; work-task rotation requires further flexibility in their job responsibilities.
- *Uncertainty tolerance*: In particular, work-related change resulting from work-task rotations or reconfigurations involves enduring change for industrial psychologists.
- *Continuous learning*: Industrial psychologists must be willing to continue learning because of frequent work-related changes.
- *Ability to work under pressure*: Shorter product life cycles and shorter marketing time mean industrial psychologists need to deal with increased pressure due to shorter product life cycles.
- *Sustainable mindset*: Industrial psychologists represent their organizations and should contribute to sustainability initiatives (Norouzi, 2022).
- *Compliance*: For instance, industrial psychologists are subject to more stringent rules regarding information technology security, machines, or hours of work (Hecklau et al., 2016).
- *Resilience*: This involves the capacity of industrial psychologists to cope despite the 4IR-STARA, barriers, or limited resources. Resilient industrial psychologists are willing and able to overcome fears of the 4IR-STARA by tapping into their emotional strength.

## Discussion and Practical Implications

### The Strategic Intelligence Role of Industrial Psychologists in the Fourth Industrial Revolution

Given the proposed competency model, industrial psychologists must perform a strategic intelligence role in organizations in terms of the top ten 4IR workplace trends identified in 2021 (Stark, 2021).

#### 4IR Trend #10: Virtual Learning

In 2021, the tenth most impactful trend was virtual learning, in which 4IR technology delivered instruction and facilitated more effective learning. The use of 4IR technology for enabling digital learning and gamification has been evolving for decades, along with the advancement of robust technology and algorithms; however, the global pandemic, where many were confined to their homes and were unable to attend traditional classrooms, accelerated usage and adoption across workplaces and educational institutions globally (Mulyadi et al., 2022). Industrial psychologists with extensive experience in learning design, delivery, and measurement developed and implemented platforms and tools. A few examples include learning experience platforms to complement traditional learning management systems, updating learning programmes to be mobile/remote-first, incorporating behavioral economics into approaches to facilitate action, and analyzing how knowledge is retained and applied differently because of new delivery methods (Boyle, 2021).

#### 4IR Trend #9: Building Cultures of Agility and Adaptability

Various industries and locations are experiencing so much disruption in 4IR that many businesses have had to adjust their business strategies and work approaches accordingly. Some organizations reduced their workforces, while others rapidly expanded, resulting in a considerable amount of change in a short time. Many companies found it easier to navigate the change than others, and some that do not describe their organization cultures as agile and adaptive have begun to build this capability going forward. To support organizations to respond, industrial psychologists apply diagnostic tools, create playbooks, and design other interventions to help them adopt new values, change their mindsets, and develop their capabilities. By leveraging data to inform decision making and updating existing practices, industrial psychologists are also helping their organizations to increase their agility and adaptability, through strategic practices such as workforce planning, talent analytics, and talent management and development (DeMeuse, 2021).

#### 4IR Trend #8: The Changing Nature of Work

Recently, many advancements in this trend can be explained by the growing adoption of artificial intelligence, increasing digitisation of processes, increasing automation, and changing approaches to who (e.g., employees, contractors, consultants) performs the work and how, which are often determined by changes in the required skill sets. Despite its influence in many domains of technology, artificial intelligence itself lacks any theory of how humans function. In the future, industrial psychologists will play a pivotal role in integrating psychological research on job performance and individual wellbeing with cutting-edge artificial intelligence techniques. By leveraging artificial intelligence in a manner that supports individuals rather than solely focusing on organizational efficiency, it is possible to strengthen the humanistic aspects of work. Artificial intelligence should be guided and steered by industrial psychologists to make the workplace a healthier one for humans (Sydell, 2021).

#### 4IR Trend #7: Work-Life Integration

As a result of the COVID-19 pandemic, work-life integration became a key trend. The shift to work from home brought together work and other aspects of life in new ways. Adaptations must be made to take care of schooling for children, managing illnesses in family and friends and other aspects such as community involvement, wellbeing, health, and other lifestyle aspects. In addition to helping organizations understand their employees' challenges and update workplace practices, industrial psychologists can assist with stress management, workplace engagement (for instance introducing stress management tools), training, flexibility, and other important interventions (McDermott, 2021).

#### 4IR Trend #6: Team Effectiveness Across Virtual and Distributed Environments

With many workers no longer working at their offices, team effectiveness re-emerged in 2021 as a top trend. Since collaboration 4IR technology developed significantly in recent years, some organizations have become accustomed to working without being physically co-located but others are dealing with productivity challenges as leaders, managers, and team members who previously relied on physical proximity are adopting new methods of working. Industrial psychologists could provide organizations with models and training on how to respond to this trend by facilitating the alignment of resources, the facilitation of effective communication, conflict management, and other effective behaviors that foster and sustain team performance (Curphy, 2021).

#### 4IR Trend #5: Social Justice

Corporate social responsibility (CSR) programmes and organizational practices have for decades incorporated elements of social justice, ensuring that all people have equal rights and opportunities regardless of individual factors. In the United States of America, when a white police officer killed George Floyd in Minneapolis, it seemed to serve as a tipping point for many organizations integrating these programmes further into their operations. Industrial psychologists assist organizations to address these complex issues by providing advice and facilitating the identification of meaningful goals that meet the ecosystem's needs and developing road maps (including leadership commitments, employee and community involvement strategies, skill-building activities, measurement tools) to address them (Beri, 2021).

#### 4IR Trend #4: Inclusive Practices to Get, Keep and Grow Talent

An inclusive culture is a practice implemented in organizations to ensure that all people feel valued and accepted in the workplace regardless of their identities (for example, race/ethnicity, gender, sexual orientation, gender identity, disability, social class, religion). Diversity in the workplace can enhance the number of positive outcomes resulting from individual and organizational diversity. Diversity, equity, and inclusion are managed effectively when diverse talent is enthusiastic about working at an organization, can put forth their best performance, and wants to stay. Industrial psychologists are uniquely qualified to assist in this effort in several ways, including providing organizations with knowledge of inclusive practices, evaluating current practices, identifying improvement areas, designing training to support implementation, exploring the role of implicit and explicit bias in organizational processes, and designing interventions to address disparities in job attitudes across groups (Jones, 2021).

#### 4IR Trend #3: Implementing Strategies and Measuring Progress on Diversity, Equity, Inclusion, and Belongingness

In contrast to the previous trend, diversity, equity, inclusion, and belongingness programmes are being measured as their impacts emerged as a separate trend in 2021. Talent analytics capabilities are constantly evolving, which is to be expected. Progress on diversity, equity, inclusion, and belongingness initiatives, or the lack thereof, has become increasingly visible in recent years thanks to the collection of accurate and consistent data over time, and the availability of practical reporting and dashboarding tools for key stakeholders. In addition to managing large amounts of information, industrial psychologists can aid organizations in providing descriptive analysis, predictive insight, and prescriptive recommendations that will lead to increased awareness and education (Cooley, 2021).

#### 4IR Trend #2: Employee Health, Wellbeing, Wellness, and Safety

Organizations continue to focus on this trend as a top priority. The cost of benefits is increasing, and organizations are investing in ways to provide employees with stress management to help reduce the physical, mental, and emotional strain that drives the increase. Costs such as lower engagement, performance, and retention are both direct to the balance sheet and indirect to the balance sheet. As a result of COVID-19, safety has become a much greater priority for essential employees and non-essential employees; employees have been working from home and experienced greater integration between work and family. By identifying root causes of stress and designing programmes to reduce risks, industrial psychologists support organizations. In collaboration with other experts, industrial psychologists advise employers and employers' expanded workforces about implementing policies and practices that promote employee health, wellbeing, wellness, and safety (Arvan and Fletcher, 2021).

#### 4IR Trend #1: Remote Work and Flexible Working Arrangements

As the leading 4IR trend in 2021, working remotely and collaborating on flexible work arrangements dominated. While the rise of remote work has different implications for different industries, occupations, and regions, it has had a broad impact across the board for both employers and employees. Industrial psychologists can play a strategic intelligence role in guiding organizations to embrace this 4IR trend by leading them to update their culture and leadership practices. By rebuilding offices into only hot desks and communal meeting areas, strategies can be less reliant on geography, while in other cases, they need to consider the effects on different types of workers. Industrial psychologists may also assist organizations with the development of remote working policies, compensation policies, productivity measures, hiring practices, satisfaction and retention assessments, and career development strategies (Wuerfel, 2021).

## Conclusion

As an industrial psychologist with experience in 4IR, STARA contexts, the author regards himself as having played an important role in the education and training of highly qualified industrial psychologists. Students who qualify can contribute competently and ethically to strategic and operational human resources (personnel) practice, as well as people (individuals, groups, organizations) behavioral dynamics, assessment, and intervention design in organizations. A generational and culturally diverse knowledge and information society demand attention to both disruptive changes in 4IR, STARA, and evolving needs. In line with the Health Professions Council of South Africa's (HPCSA) scope of practice for industrial psychologists, and the HPCSA Minimum Standards for the Training of Industrial Psychology (February 2019), the curriculum is designed to focus on the development and application of industrial and organizational psychology domain competencies pertaining to tangible and observable human behavior-related diagnosis, design, intervention and assessment applied at the individual, group and organizational levels. To understand, modify and enhance individual, group and organizational behavior wellbeing and effectiveness (Coetzee and Oosthuizen, 2019), and to enhance individual, group and organizational wellbeing, the objectives of the training include the planning, development, and application of universal, Afrocentric, and psychological paradigms, theories, models, models, constructs, and principles.

The industrial psychology profession must contribute to the development, design, and implementation of methods of inquiry that utilize specialized knowledge, skills, and technologies that are relevant to the profession's ability to respond to complex and challenging human behavior issues within a 4IR organizational context. The solutions, insights, and new knowledge that can be generated by advanced scholarship and research in the South African and African work contexts may contribute to productivity, human growth, and quality of life at work. Interns pursuing further distance education and training as prospective industrial psychologists can gain practical experience through open distance education and learning. Training and education for industrial psychologists are based on experiential learning involving the application of knowledge the article concluded with an analysis of the various ramifications of the 4IR, STARA, particularly for industrial psychologists. STARA will forever change the profession of industrial psychology, and current industrial psychologists should take this into consideration. As a result, they need to gradually become aware of the potential impacts of STARA inside their field and design strategies for how to cope with them. In an age where AI and machine learning are becoming more common, industrial psychologists can play a vital role in ensuring the effective use of information and research, assisting with data translation, and ensuring the legitimate reliability of information models and their use. By creating interventions to enable employees to adjust to AI “colleagues”, industrial psychologists could assist researchers in understanding their employees' reactions to AI. The inclusion of industrial psychologists in information technology departments will be a basic requirement for organizations to use their skills, ensuring optimal results (SIOP Communications Department the Media Subcommittee of SIOP's Visibility Committee, 2019). As an opening to research and self-awareness, this new period of the 4IR could be viewed as a time of energy for industrial psychologists. Industrial psychologists play a strategic intelligence role in assisting organizations with disruptive change and development through the provision of wellness programmes to assist employees to cope with these changes (Coetzee and Oosthuizen, 2019).

## Data Availability Statement

The raw data supporting the conclusions of this article will be made available by the author, without undue reservation.

## Author Contributions

RO conceptualized and wrote the article.

## Conflict of Interest

The author declares that the research was conducted in the absence of any commercial or financial relationships that could be construed as a potential conflict of interest.

## Publisher's Note

All claims expressed in this article are solely those of the authors and do not necessarily represent those of their affiliated organizations, or those of the publisher, the editors and the reviewers. Any product that may be evaluated in this article, or claim that may be made by its manufacturer, is not guaranteed or endorsed by the publisher.
